# Optimizing an avian influenza vaccine using a novel Bacterial Enzymatic Combinatorial Chemistry (BECC) TLR4 adjuvant

**DOI:** 10.1128/msphere.00171-26

**Published:** 2026-07-10

**Authors:** Devon V. Riley, Lauren Baracco, Sayan Das, Brandon M. Tenaglia, Sydney Speed, Carly Dillen, Juliahna Hayes, Samanta Del Veliz, Haye Nijhuis, Valerie Le Sage, Hannah W. Despres, Lynda Coughlan, Weina Sun, Matthew B. Frieman, Robert K. Ernst

**Affiliations:** 1 Department of Microbiology and Immunology, School of Medicine, University of Maryland, Baltimore, Maryland, USA; 2 Department of Microbial Pathogenesis, School of Dentistry, University of Maryland, Baltimore, Maryland, USA; 3 Center for Vaccine Development and Global Health (CVD), University of Maryland School of Medicine, Baltimore, Maryland, USA; 4 Center for Vaccine Research, University of Pittsburgh School of Medicine 12265, Pittsburgh, Pennsylvania, USA; 5 Department of Microbiology and Molecular Genetics, University of Pittsburgh School of Medicine, Pittsburgh, Pennsylvania, USA; 6 Center for Pathogen Research, Department of Microbiology and Immunology, University of Maryland School of Medicine, Baltimore, Maryland, USA; 7 Department of Microbiology at Icahn School of Medicine at Mount Sinai, New York, New York, USA; University of Missouri, Columbia, Missouri, USA

**Keywords:** avian viruses, influenza vaccines, adjuvants

## Abstract

The development of broadly protective and dose-sparing influenza vaccines remains a critical challenge, particularly for zoonotic H5N1 strains with pandemic potential. This study evaluates BECC470s, a synthetic TLR4 adjuvant, for its ability to enhance the immunogenicity and protective efficacy of recombinant H5 hemagglutinin (rHA) vaccination in murine models. BECC470s-adjuvanted rHA elicited robust IgG1/IgG2a antibody responses and complete survival following homologous 2004 H5N1 challenge in a prime–boost model. Although BECC470s broadened antibody binding to both variable HA head and conserved stalk domains by ELISA, functional neutralizing antibody responses were restricted to the matched 2004 H5N1 isolate, with no detectable neutralization of H5N1 viruses isolated in 2022 or 2024. These data indicate that BECC470s enhances the magnitude and apparent cross-reactivity of binding antibody responses while maintaining strain-specific neutralizing activity, supporting its potential as an adjuvant for next-generation influenza vaccines while underscoring the need for further optimization to achieve true cross-neutralizing protection.

**IMPORTANCE:**

BECC470s is a synthetic TLR4 agonist adjuvant that enhances the breadth and potency of recombinant H5 hemagglutinin vaccination in mice. By driving balanced antibody responses, complete protection against homologous challenge, and neutralizing activity targeting both HA head and stalk, BECC470s outperforms the comparator TLR4 adjuvant. These preliminary findings indicate that BECC470s enables antigen sparing while increasing antigen immunogenicity, supporting its potential translational application to current H5N1 vaccines.

## INTRODUCTION

Influenza virus infection remains a major global public health challenge. In the United States, the CDC estimates that, between 2010 and 2024, seasonal influenza has resulted annually in approximately 9.3 to 41 million illnesses, 120,000 to 710,000 hospitalizations, and 6,300 to 52,000 deaths (https://www.cdc.gov/flu-burden/php/about/index.html). The resulting hospitalizations and morbidity imposed a substantial economic burden through both lost productivity and increased healthcare costs. While annual influenza epidemics are primarily driven by seasonal influenza A and B viruses, highly pathogenic avian influenza (HPAI) viruses, such as H5 and H7, occasionally infect humans, occasionally infect humans, and continue to pose a persistent zoonotic and pandemic threat. These viruses are particularly concerning due to their potential to cause severe disease, with mortality rates exceeding 50% during some outbreaks (https://www.cdc.gov/bird-flu/situation-summary/wildbirds.html). Developing effective human avian influenza virus vaccines is essential to address this gap and reduce the risks associated with emerging zoonotic and pandemic strains. Current vaccines for avian influenza virus in humans, especially those based on the H5 subtype of the hemagglutinin (HA) protein, exhibit substantial limitations. These vaccines frequently exhibit low immunogenicity, requiring large antigen doses (up to 90 µg per dose) or multiple booster immunizations to elicit protective antibody responses (1–5), while the basis for this limited immune response remains unclear.

Generating antibodies directed toward both the HA head and stalk domains of influenza A virus (IAV), along with targeting key antigenic sites on H5 HA, is critical due to their complementary roles in virus neutralization and cross-protection even though antibody responses typically focus on the HA head. HA1 contains the globular head domain, which is highly variable between subtypes, and HA2, which consists of stalk and a small part of HA1 (6) and is more conserved between subtypes. Antibodies directed against the HA head domain inhibit the interaction between viral particles and sialic acid receptors (7), thereby playing a pivotal role in the neutralization of specific influenza virus strains; whereas those recognizing the conserved HA stalk domain contribute to broad cross-protective immunity, underscoring the necessity to generate both antibody subsets in vaccination strategies.

Adjuvants offer a promising strategy to overcome these limitations by not only enhancing the magnitude of the immune response but also modulating its qualitative aspects by shaping the nature and durability of the immune response through immunomodulatory mechanisms (8). In particular, adjuvants that activate innate immune receptors, such as Toll-like receptors (TLRs), can drive potent and durable humoral and cellular immunity (9, 10). BECC470s is a rationally engineered synthetic Toll-like receptor 4 (TLR4) ligand derived through bacterial enzymatic combinatorial chemistry (BECC). It is designed to activate innate immunity by mimicking and modifying known lipid A structures, thereby eliciting a balanced and potent immune response to enhance vaccine efficacy (11).

Building on our previous findings that BECC470s enhances immune responses to trimeric H1 HA (12–14), our current results demonstrate that BECC470s is a potent adjuvant that can also significantly enhance the immunogenicity of the H5 HA antigen, resulting in improved antibody responses and increased protective efficacy at low vaccine doses. Here, we report that BECC470s augments the protective efficacy of a recombinantly produced H5 HA (rHA) derived from A/Vietnam/1203/2004 (H5N1) by enhancing humoral responses and broadening epitope recognition. Using a murine challenge model, mice that received a single prime immunization of BECC470-adjuvanted rHA were effectively protected against IAV-associated morbidity and mortality following a homologous IAV challenge with a 6:2 PR8 reassortant virus with low pathogenicity HA (polybasic cleavage site removed: HALo) from the A/Vietnam/1203/2004 strain (15) (hereafter referred to as PR8/H5). Mechanistically, BECC470s broadened HA domain targeting and potentiated neutralizing antibody responses, with a notable increase in linear B cell epitope-specific antibodies focused on the mapped antigenic site B on the H5 HA protein. In addition, vaccination with H5 HA and BECC470s induced antibodies that bound a wide range of HAs from phylogenetically divergent influenza virus strains. These findings demonstrate the capacity of BECC470s to enhance both the extent and potency of cross-reactive antibody responses essential for improved influenza vaccination strategies. Given the continuous emergence of novel influenza strains, the development of adjuvanted vaccines capable of eliciting broad and robust protection is imperative for pandemic preparedness and global health security.

## RESULTS

### BECC470s adjuvant enables dose-sparing and enhances rHA-mediated protection against homologous IAV after prime-boost vaccination

To evaluate the immunogenicity and protective efficacy, recombinant hemagglutinin (rHA) vaccine formulations adjuvanted with the novel TLR4 agonist BECC470s or the MPLA-mimetic PHAD were tested in a prime-boost mouse model of homologous influenza A virus challenge. We assessed the immune responses and protective outcome in mice vaccinated with rHA in combination with either BECC470s or PHAD, a synthetic TLR4 agonist structurally related to detoxified monophosphoryl lipid A (MPLA) (16).

**Fig 1:**
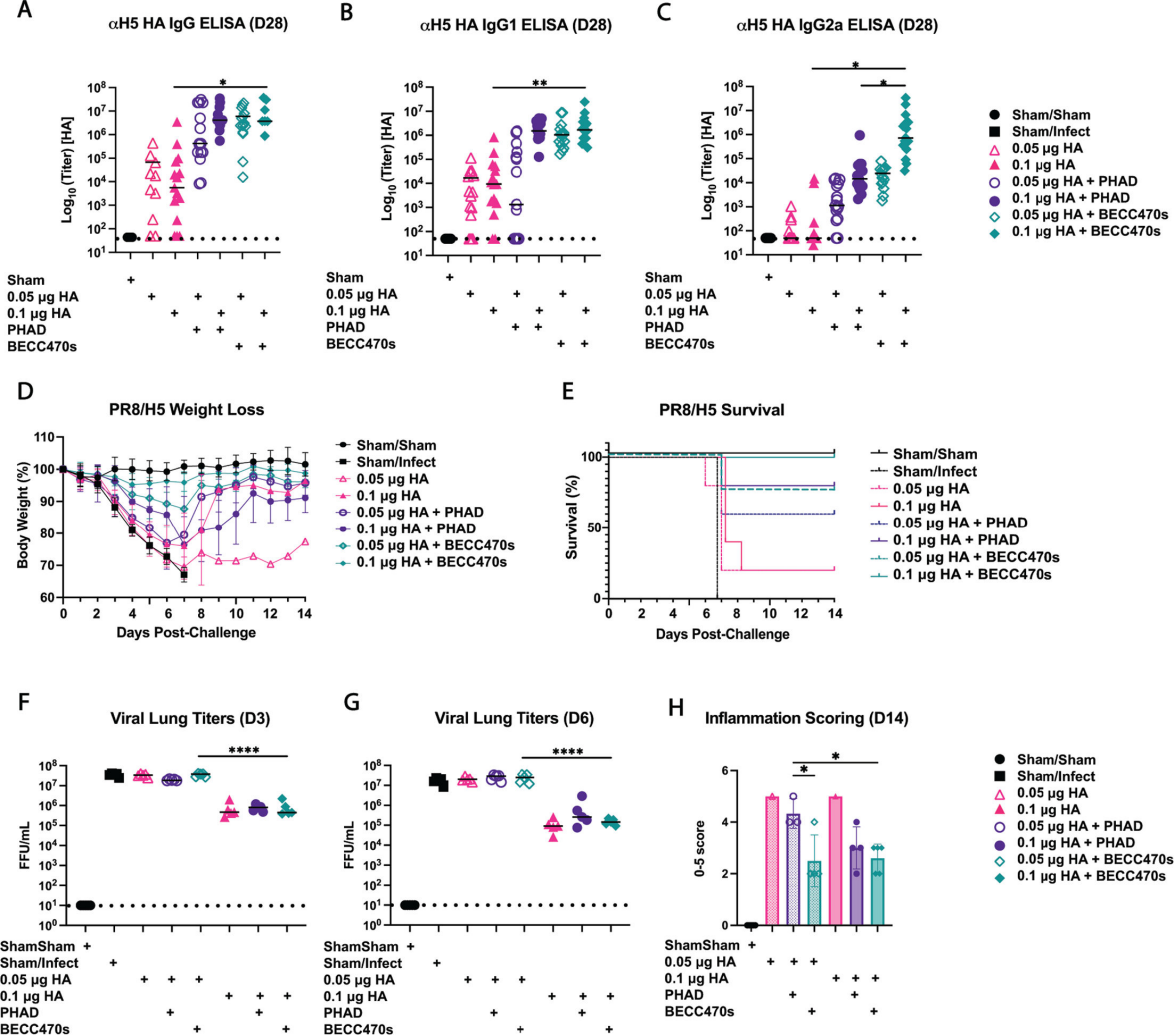
Determining rHA dose needed in a prime-boost vaccination model for protection against homologous IAV challenge. Six-week-old BALB/c mice (*n* = 15) were immunized via a prime-boost regimen (days 0 and 14) with 0.05 μg or 0.1 μg rHA derived from A/Vietnam/1203/2004, formulated either without adjuvant, with 50 μg PHAD, or with 50 μg BECC470s preceding PR8/H5 challenge (500 PFU) at day 28. (**A**) Pre-infection day 28 serum ELISA total IgG, IgG1 (**B**) and IgG2a (**C**) (*P < 0.02, **P < 0.008, ****P < 0.0001). (**D**) Fourteen-day weight loss in BALB/c mice (*n* = 15) and (**E**) 14-day survival graph. (**F**) Virus titer of lung homogenate 3 days post infection and (**G**) 6 days post infection. (**H**) Averaged alveolar and peribronchiolar scoring of lung histology slides 14 days post-infection. BECC470s-adjuvanted rHA vaccine protects mice following a single prime dose.

In this study, PHAD, a synthetic TLR4 agonist, was used as a comparator as it mimicked the immunostimulatory effects of monophosphoryl lipid A (MPLA) while offering improved reproducibility, lower toxicity, and easier manufacturing compared with naturally derived MPLA preparations. By directly comparing BECC470 to PHAD, we benchmark our novel TLR4-based adjuvant against a well-characterized MPLA mimetic to assess relative potency, immune polarization, and translational potential, given that MPLA-like TLR4 agonists are already used in licensed human vaccine adjuvants (17, 18). To determine the minimum concentration of rHA required to confer protection against homologous IAV challenge, a concentration-response experiment was conducted using a prime-boost vaccination regimen (days 0 and 14) with or without adjuvants. Sera were collected at day 28 post vaccination, and immunogenicity was assessed by quantifying total IgG, IgG1, and IgG2a titers via ELISA (Fig. 1A through C). BECC470s demonstrated antigen-sparing, as total IgG responses were similar between 0.05 µg and 0.1 µg rHA doses; however, the 0.1 µg rHA + BECC470s group elicited significantly higher IgG levels than 0.1 µg rHA administered without adjuvant (*P < 0.0117; Fig. 1A). In mice receiving BECC470s as an adjuvant, IgG1 titers reached saturation at both concentrations, whereas PHAD required higher rHA doses to achieve comparable levels. The 0.1 µg rHA + BECC470s-adjuvanted group exhibited significantly higher IgG1 titers than 0.1 µg rHA alone (**P < 0.08; Fig. 1B). Notably, only the 0.1 µg rHA + BECC470s group generated significantly elevated IgG2a titers compared with unadjuvanted 0.1 µg rHA or 0.1 µg rHA + PHAD (*P < 0.02), consistent with a more balanced Th1/Th2 response (Fig. 1C).

On day 28, vaccinated and sham control BALB/c mice (*n* = 15 per group) were challenged intranasally with 500 PFU of PR8/H5 and monitored daily for weight loss (Fig. 1D). Survival was assessed over 14 days post-challenge (Fig. 1E). Both unadjuvanted rHA doses failed to protect mice from weight loss, with only 20% survival in these groups. In comparison, formulations containing BECC470s provided nearly complete protection against weight loss, and the group receiving 0.1 µg rHA with BECC470s achieved total survival (Fig. 1E). Viral titers in lung homogenates collected on days 3 and 6 post-challenge were significantly reduced in the group vaccinated with 0.1 µg rHA compared to the 0.05 µg rHA group (****P < 0.0001) (Fig. 1F and G).

Histological analysis of lung tissues stained with hematoxylin and eosin (H&E) to visualize tissue structure at day 14 post-challenge revealed significant differences in both peribronchiolar and alveolar inflammation among treatment groups (Fig. S1). While the 0.05 µg and 0.1 µg rHA vaccine groups exhibited bronchiolar and periarterial inflammation, both BECC470s-adjuvanted groups showed significantly lower inflammation scores (*P < 0.05) compared to the 0.05 µg rHA + PHAD group. Despite similar lung viral titers observed between the 0.05 µg and 0.1 µg rHA groups, inflammation at day 14 differed markedly, with the BECC470s-adjuvanted groups demonstrating the lowest inflammatory cell infiltration, with scores approaching baseline levels (Fig. 1H). Mice receiving PHAD as an adjuvant exhibited intermediate inflammation levels. Collectively, vaccination with 0.1 µg rHA combined with BECC470 significantly mitigated virus-induced lung inflammation, indicating enhanced protective potential relative to alternative vaccine formulations. These results demonstrate that BECC470s is a highly effective adjuvant that enhances immune responses, supports antigen dose sparing, and provides complete protection against homologous IAV challenge in a prime-boost vaccination model.

### BECC470s-adjuvanted rHA vaccine protects mice following a single prime dose

In the United States, FDA-approved annual influenza vaccines are administered as a single dose for adults or 1 to 2 doses for pediatric patients and are formulated as trivalent preparations containing three distinct hemagglutinin (HA) antigens (https://www.fda.gov/vaccines-blood-biologics/influenza-vaccine-composition-2025-2026-us-influenza-season). The standard adult dose includes 15 µg of HA per viral strain in the vaccine (https://www.cdc.gov/flu/professionals/acip/app/dosage.htm). Ideally, a single immunization would elicit robust titers of influenza-specific antibodies that confer protective immunity. A single-dose immunization capable of inducing strong immunity is highly advantageous during a pandemic, as it enables rapid population coverage, enhances vaccine uptake, and reduces financial and logistical burdens. With repeated vaccination, immune boosting effects occur with each dose as memory B cells recall previous exposures; however, after initial vaccination, older adults generally produce only modest levels of antibodies (19–21).

**Fig 2:**
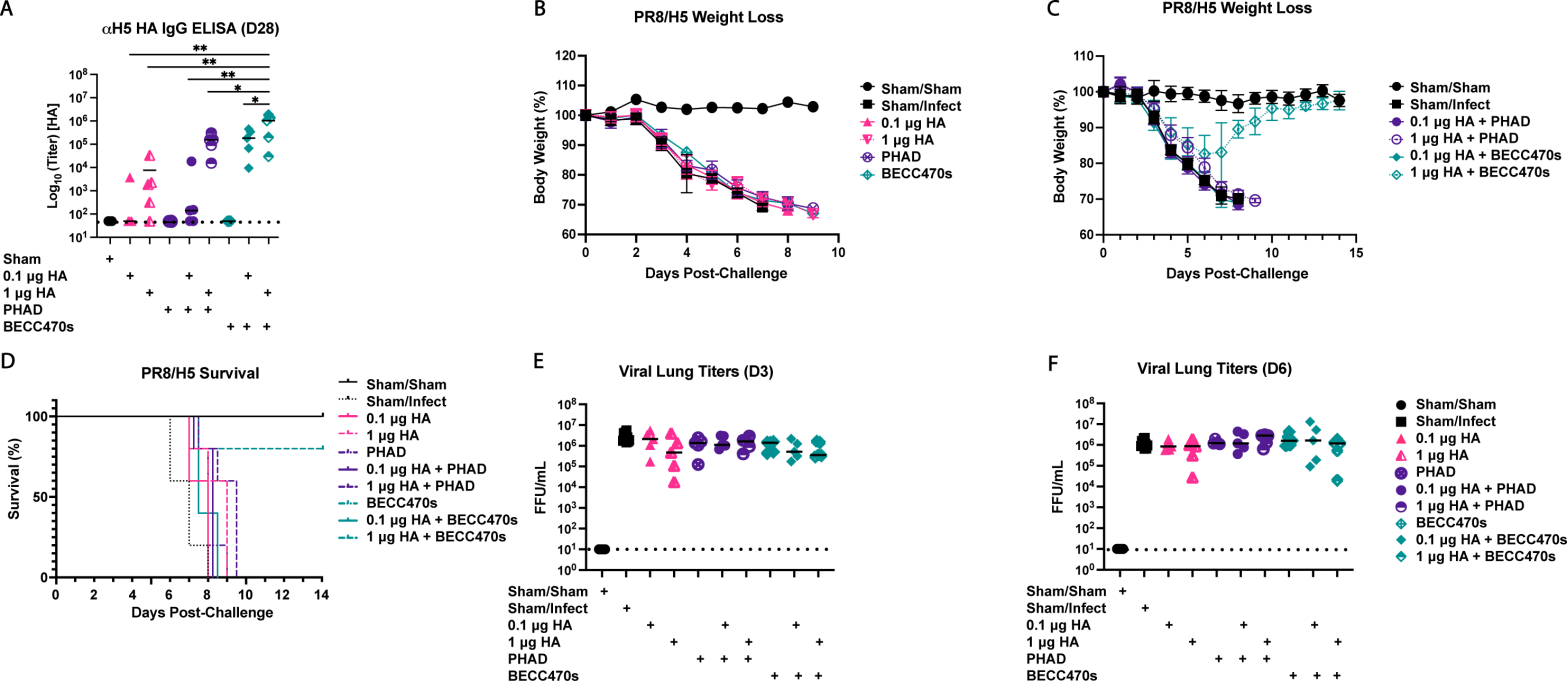
Prime-only BECC470s-adjuvanted rHA vaccine confers protection in mice. (**A**) Pre-infection day 28 serum ELISA of total IgG with vaccination including 0.1 or 1 μg HA protein in combination with 50 μg of BECC470s or PHAD adjuvants in prime only vaccination schedule (*P < 0.05, **P < 0.005). (**B** and **C**) Fourteen-day weight loss in BALB/c mice (15 per group) after infection with 500 PFU of PR8/H5 with (**D**) 14-day survival graph. (**E**) Virus titer of lung homogenate 3 days post infection and (**F**) 6 days post infection.

To evaluate the efficacy of a prime-only adjuvanted vaccine against homologous influenza A virus (IAV) challenge, BALB/c mice (*n* = 15 per group) were immunized with 0.1 or 1 µg of recombinant HA (rHA), alone or in combination with 50 µg of PHAD or BECC470s. A dose of 0.1 μg rHA combined with BECC470s was previously established as protective in a prime-boost model (Fig. 1). Antibody responses were assessed in each vaccination group to correlate with observed *in vivo* protection. Total serum IgG levels were quantified by ELISA at day 28 following immunization. The mean antibody titer for mice given 0.1 µg rHA protein alone was at the limit of detection, while those receiving 1 µg rHA alone exhibited a mean titer of approximately 10^4^. Total serum IgG measured showed significantly elevated titers in the 1 µg + BECC470s group compared to all other groups, including PHAD-containing formulation (*P < 0.05, **P < 0.005; Fig. 2A).

Mice receiving unadjuvanted rHA or adjuvant alone showed no protection from weight loss post-challenge, losing ~30% of their starting weight (Fig. 2B). Only the 1 µg rHA + BECC470s group demonstrated protection from ~18% wt loss (Fig. 2C), as well as protection from mortality, with 80% survival observed in this group (Fig. 2D); all other groups required euthanasia by day 9 due to IACUC protocol regulations for weight loss.

Lung homogenates from mice collected on days 3 and 6 post-challenge were analyzed for viral load using focus-forming unit (FFU) assays. Interestingly, no differences in lung viral load were observed among groups at either day 3 or day 6 post-infection (Fig. 2E and F) despite differences in clinical protection. These results demonstrate that BECC470s effectively enhances protective immunity in a prime-only influenza vaccination model, conferring significant survival benefit despite no measurable differences in viral lung load.

### BECC470s adjuvant broadens HA domain targeting and enhances the magnitude and potency of cross-reactive neutralizing antibodies against influenza A

**Fig 3:**
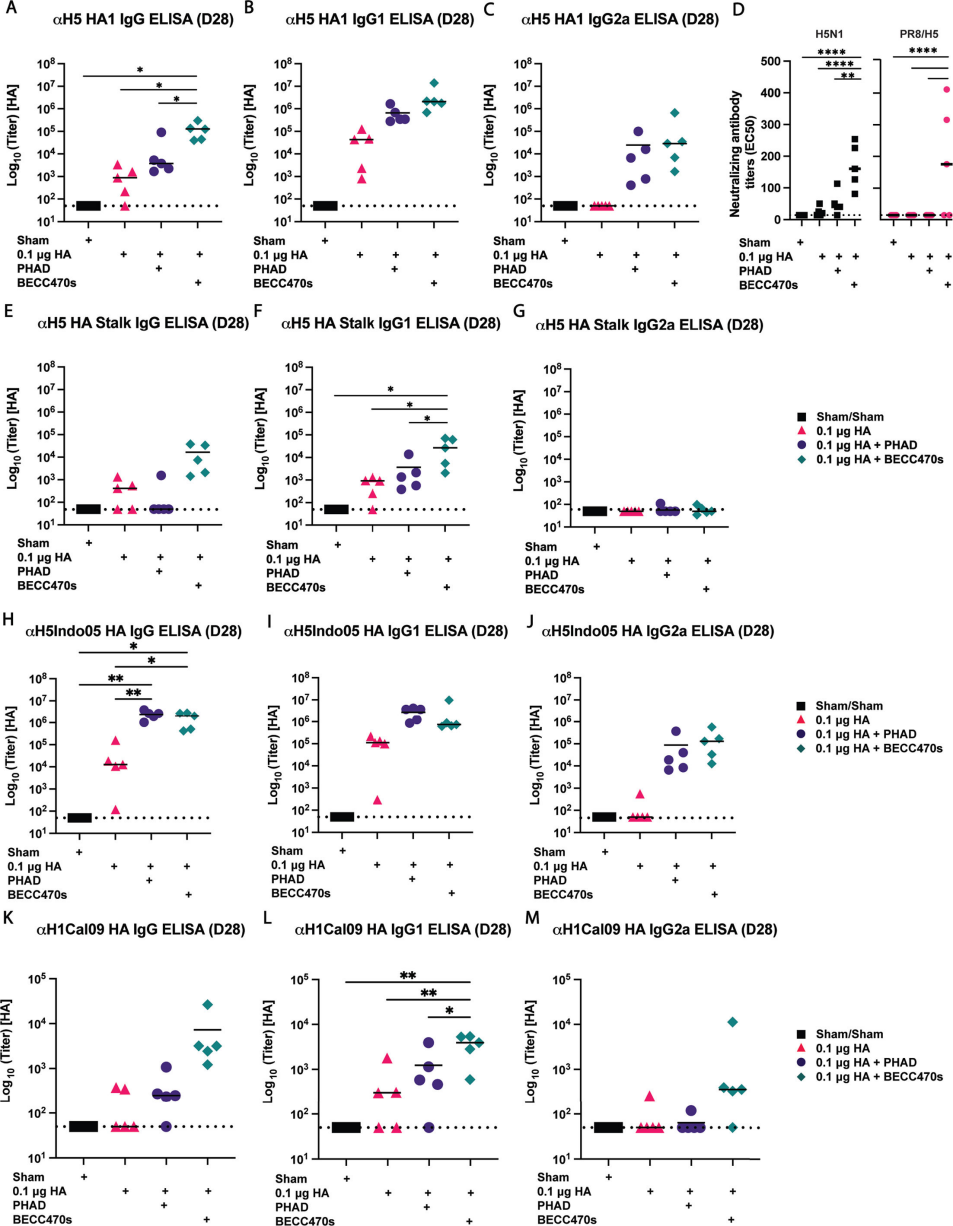
HA1, stalk-specific, neutralizing, and cross-reactive antibody responses elicited by H5 rHA vaccination in adult mice. Six-week-old BALB/c mice were immunized via a prime-boost regimen with 0.1 μg rHA derived from A/Vietnam/1203/2004, formulated without adjuvant, with 50 μg PHAD, or with 50 μg BECC470s. Day 28 post-immunization sera were collected, and antibody titers specific for αH5 HA1 were quantified by ELISA; total IgG (**A**), IgG1 (**B**), and IgG2a (**C**) levels were measured (*P < 0.05). (**D**) Neutralizing activity was evaluated by microneutralization assay against PR8/H5 (pink circles, right) and A/Vietnam/2004 (H5N1; black squares, left) viruses. The horizontal dashed line denotes the assay limit of detection (LOD). Antibody titers specific for αH5 stalk were quantified by ELISA; total IgG (**E**), IgG1 (**F**), and IgG2a (**G**) levels were measured (*P < 0.05). Antibody titers specific for αH5 or H1 HA were quantified by ELISA using A/Indonesia/5/2005 HA (**H–J**) or A/California/07/2009 HA (**K–M**) as the coating antigen. Total IgG, IgG1, and IgG2a were measured for each coating antigen. (*P < 0.02, **P < 0.009, ****P < 0.0001). Prism 10 was used for statistical comparisons between using two-way ANOVA with multiple comparisons.

To evaluate the contribution of specific hemagglutinin regions to antibody cross-reactivity, ELISA assays were performed using antigens derived from either the HA1 domain or the conserved HA stalk. This approach allowed for a comparative analysis of antibody responses specific to each domain, thereby clarifying their respective roles in cross-reactive recognition. To determine if adjuvanting H5 HA with BECC470s increased cross-protective immunity, we analyzed the HA1 and HA stalk binding capability of antibodies from rHA-vaccinated mice vaccinated with either protein alone, rHA + PHAD or rHA + BECC470s and showed that serum antibodies from mice receiving a prime-boost regimen demonstrated that addition of BECC470s as an adjuvant enhanced HA-specific humoral responses. Specifically, BECC470s-adjuvanted vaccines elicited significantly greater titers of both αH5 HA1 (Fig. 3A) and αH5 stalk (Fig. 3E) total IgG by day 28 post-prime compared to non-adjuvanted and PHAD groups (*P < 0.05). These elevated titers were predominantly due to increased IgG1 subclass responses (Fig. 3B and F), while IgG2a responses were generally lower and did not differ significantly among groups (Fig. 3C and G). Vaccination with rHA alone or with PHAD resulted in minimal stalk-binding antibodies, whereas BECC470s conferred a robust IgG1 response specific to the conserved HA stalk domain.

Binding antibodies, quantified by ELISA above, demonstrate immunogenicity but do not necessarily indicate protective efficacy. The gold-standard correlate for protection is the generation of neutralizing antibodies, as these directly inhibit viral entry and replication in host cells (22, 23). The ability of neutralizing antibodies to target influenza virus surface proteins, such as hemagglutinin, plays a fundamental role in preventing infection and limiting disease severity, underscoring their significance in both natural immunity and vaccine-induced protection (24, 25). Microneutralization (MN) assays were performed to examine adjuvant-dependent enhancement of neutralizing antibody responses using microneutralization assays against PR8/H5 (A/Vietnam/120/3/2004 HALo) or a wild-type A/Vietnam/2004 isolate (performed in BSL-3 containment). Mice immunized with BECC470s-adjuvanted H5 rHA exhibited significantly higher neutralizing antibody titers against PR8/H5 demonstrating strong inhibition for the homologous strain (****P < 0.0001 compared to all other groups; Fig. 3D). To assess reactivity against H5N1 strains at BSL-3, live virus microneutralization assays were conducted against A/Vietnam/2004, as well as two recently emergent strains, A/dairy cattle/Texas/24008749001/2024 (H5N1) and A/mink/Spain/3691 8_22VIR10586-10/2022 (H5N1). While robust strain-specific neutralizing titers were observed against the original H5N1 strain in BECC470s-adjuvanted vaccinated group only (**P < 0.02, ****P < 0.0001 compared to all other groups; Fig. 3D), no neutralizing antibodies were detected against the cow- and mink-derived viruses (Fig. S2). In contrast, sera from mice immunized with rHA alone, PHAD-adjuvanted vaccine, or sham controls showed neutralizing titers at or below the assay detection limit for all tested viruses. Although PHAD modestly enhanced responses relative to unadjuvanted rHA, BECC470s outperformed PHAD in eliciting potent neutralizing antibodies against homologous H5N1 viruses. To evaluate whether vaccine-induced antibodies could neutralize a heterosubtypic strain, sera were tested against A/Netherlands/602/2009 (H1N1), and no detectable neutralizing activity was observed in any vaccination group (Fig. S3).

To evaluate potential cross-reactivity against more diverse HA subtypes, ELISAs against rHA from either A/Indonesia/5/2005 (H5N1) or A/California/7/2009 (H1N1) were used. The A/Indonesia/5/2005 HA shares 97% sequence homology with the immunizing A/Vietnam/1203/2005 HA, while the A/California/7/2009 HA is more divergent, sharing 63% sequence homology; all HA sequences were aligned and analyzed using Geneious Prime version 2025.2.2.

Mice immunized with rHA alone generated low but detectable αH5 and αH1 IgG titers, whereas PHAD significantly boosted total and IgG1 responses and BECC470s further increased titers by 1–2 orders of magnitude across both specificities (*P < 0.02, **P < 0.009; Fig. 3H through M). Both adjuvants enhanced IgG1, but BECC470s consistently elicited the highest levels and uniquely drove a marked increase in IgG2a, indicative of enhanced cross-reactive potential. These data show that BECC470s-adjuvanted H5 rHA induces robust heterologous and heterosubtypic antibody responses, supporting its promise as an adjuvant for broad protective immunity against diverse influenza A viruses.

Collectively, these findings demonstrate that BECC470s-adjuvanted H5 rHA elicits robust cross-reactive heterologous and heterosubtypic antibodies together with potent strain-specific neutralization, highlighting BECC470s as a highly effective adjuvant for broad protective humoral immunity targeting both conserved and variable HA domains across diverse influenza A virus strains.

### Enhanced magnitude of antibody responses to antigenic site B induced by BECC470s-adjuvanted rH5 HA vaccination

**Fig 4:**
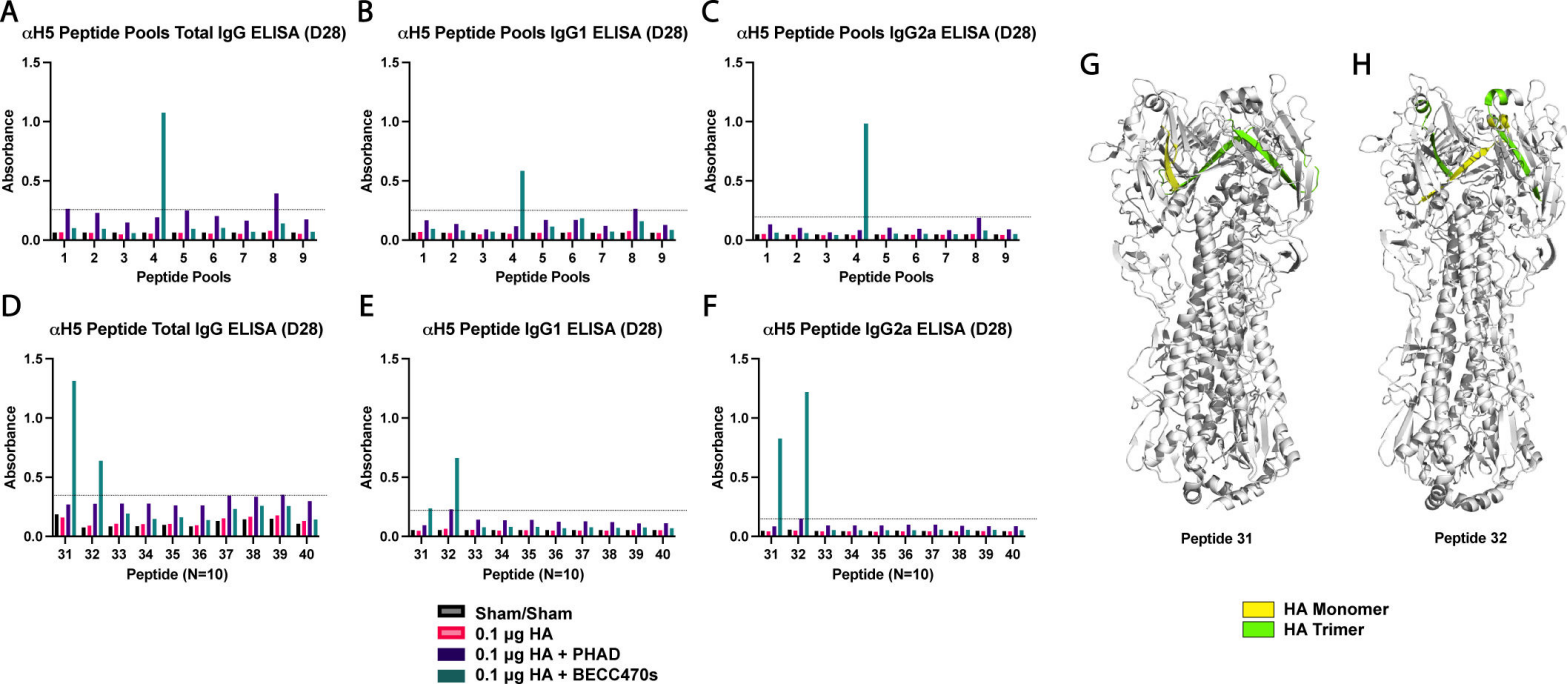
BECC470s-adjuvanted immunization broadens linear B cell epitopes. Six-week-old BALB/c mice were immunized via a prime-boost regimen with 0.1 μg rHA derived from A/Vietnam/1203/2004, formulated without adjuvant, with 50 μg PHAD, or with 50 μg BECC470s. Initially, 93 overlapping peptides (12- or 17-mers with 11 amino acid overlaps) spanning the H5 HA of A/Vietnam/1203/2004 were pooled into nine pools. Pre-infection sera, day 28 post-immunization, were collected and total IgG (**A**), IgG1 (**B**), and IgG2a (**C**) titers against peptide pools were determined by ELISA. Individual peptides for pool 4 was tested with total IgG (**D**), IgG1 (**E**), and IgG2a (**F**) determined by ELISA. The dashed line indicates an O.D. value fourfold higher than that of the sham control group; samples above this threshold are considered positive. Ribbon diagrams depicting a 3D schematic representation of peptides 31 (**G**) and 32 (**H**) created using PyMOL (HA monomer highlighted in yellow and HA trimer highlighted in green).

The broader protection observed with rHA formulated with BECC470s is hypothesized to result from both increased serum antibody levels and the induction of antibodies targeting diverse epitopes on the rHA protein. To define epitope-specific responses and quantify serum antibody magnitude post-vaccination, total IgG, IgG1, and IgG2a titers were measured against a panel of 93 overlapping peptides (12- or 17-mers with 11-amino acid overlap) spanning the H5 HA of A/Vietnam/1203/2004, following the method outlined by Das et al. (14). Six-week-old BALB/c mice were immunized on a prime-boost schedule with 0.1 μg rHA alone or combined with 50 μg PHAD or BECC470s. Serum samples collected on day 28 following immunization, prior to challenge, were assessed by ELISA for peptide-specific antibody binding (Fig. 4). Total IgG (Fig. 4A), IgG1 (Fig. 4B), and IgG2a (Fig. 4C) titers against individual peptide pools were determined by ELISA. Of the 93 tested peptide pools, positive antibody reactivity was detected in pool 4 (peptides 31–40) and pool 8 (peptides 72–82), with positivity defined as an O.D. value four times greater than the mean of sham-immunized controls. Pools 4 and 8 were further separated into their constituent 10 or 11 individual peptides, respectively, and assessed for antibody binding. Peptides 72–82 showed no reactivity in any group, with O.D. readings for total IgG, IgG1, and IgG2a remaining below the fourfold threshold relative to naïve controls (Fig. S4).

Mice immunized with rHA + BECC470s exhibited significantly elevated total IgG titers (Fig. 4D) against peptides 31 and 32, which correspond with antigenic site B on the HA protein (26, 27) with O.D. readings above the threshold defining positive antibody binding (dashed line). Consistent with total IgG findings, IgG subclass evaluation demonstrated that BECC470s-vaccinated mice displayed the highest IgG1 titers (Fig. 4E) toward peptides 31 and 32, reflecting a Th2-biased antibody response centered on antigenic site B. Similarly, IgG2a titers (Fig. 4F) were predominantly elevated against peptide 31, indicating a concurrent Th1-type response. In contrast, PHAD-adjuvanted and antigen-alone immunizations elicited weaker, more limited peptide-specific responses, rarely surpassing the threshold. These results demonstrate that formulation with BECC470s adjuvant significantly amplifies and broadens antibody responses targeting key linear epitopes, especially those spanning antigenic site B (peptides 31 and 32) of the H5 HA. These findings suggest that BECC470s may potentiate protective efficacy by focusing immune recognition on critical antigenic regions of the hemagglutinin protein.

## DISCUSSION

Influenza A (H5N1) remains a persistent pandemic threat, necessitating the development of innovative vaccines that extend beyond the limited efficacy of current licensed formulations. Traditional influenza vaccines are often tailored to specific strains and require annual updates, leading to immunity gaps as the virus continues to evolve. The development of cross-reactive influenza vaccines is crucial for achieving broader, longer-lasting protection, minimizing the impact of viral diversity, and improving pandemic preparedness. Antigenic drift and shift in influenza viruses drive recurrent seasonal epidemics and occasional pandemics. Cross-reactive vaccines, by targeting more conserved viral components, have the potential to maintain immunity across multiple strains and epidemic seasons, reducing the impact of antigenic variability. Current FDA-approved seasonal influenza vaccines are typically trivalent, including HAs from A (H1N1), A (H3N2), and B (B/Victoria-lineage); previously, FDA-approved quadrivalent vaccines also incorporated a second B-lineage (B/Yamagata-lineage) HA (28).

Although seasonal influenza vaccines are designed to protect against circulating human strains, studies indicate they elicit limited cross-reactive antibody responses to influenza A subtypes not included in the formulation. This restricted breadth of reactivity is particularly evident when evaluating responses across phylogenetically distinct HA groups. Specifically, minimal cross-reactivity toward group 2 influenza A viruses highlights their inability to induce broadly neutralizing antibodies, therefore limiting their potential for universal protection against emerging pandemic and avian strains (29).

These limitations highlight similar challenges in subtype-specific vaccines, including H5N1, for combating emerging influenza strains. Although existing FDA-approved H5N1 vaccines are safe and stimulate subtype-specific immunity, their limited broad coverage and lack of dose-sparing strategies pose hurdles for rapid, large-scale pandemic response (30). Currently, three H5N1 vaccines have FDA approval, with two using adjuvants (MF59 or AS03) to boost immunogenicity (https://www.fda.gov/vaccines-blood-biologics/vaccines/vaccines-licensed-use-united-states). Given this limited landscape, evaluating the efficacy of these available vaccines relies on established immunological endpoints. For these vaccines, the primary study endpoints to evaluate efficacy are hemagglutination inhibition (HI) antibody titers ≥ 1:40. While this is a standard goal of immunogenicity in influenza vaccine studies, HI titers do not always correlate fully with *in vivo* protection in animal models and do not measure other potential immune mechanisms such as antibody-dependent cellular cytotoxicity or T cell responses (31).

Together, these limitations in current vaccine evaluation and performance underscore the need to explore adjuvant strategies that not only enhance immunogenicity but also broaden protective immune responses beyond conventional correlates of protection. This study shows that BECC470s-adjuvanted rHA vaccines significantly improved survival and immunogenicity, even with reduced antigen doses or single-dose priming, while lung viral titers remained similar across adjuvanted groups, consistent with prior H5N1 studies. In addition to the immunogenicity and survival benefits, BECC470s reshape HA-specific antibody profiles to overcome structural barriers to cross-protection, specifically to the head and stalk regions of HA. Structural diversity in the HA head limits cross-subtype immunity (32), whereas conserved stalk motifs elicit broadly reactive antibodies (33, 34). Specifically, BECC470s enhanced overall titers while expanding both head- and stalk-directed responses, where head-specific antibodies provide potent but narrow neutralization and stalk antibodies engage FcγRs to mediate cytotoxic clearance (35) and broaden recognition across group 1 and 2 HAs. BECC470s further enhanced responses to antigenic site B, a key target of neutralizing antibodies and antigenic drift, resulting in strong homologous neutralization and protection, although the linear peptide assay likely underrepresents antibodies recognizing conformational epitopes. Robust neutralization of the homologous H5N1 but not recent isolates likely reflects antigenic divergence (only ~70% identity in the antigenic site B) rather than an intrinsic limitation of BECC470s, motivating future studies with targeted site B mutations, and underscoring the need for adjuvants that enhance immunity to both variable head sites (A–E) and conserved regions (26, 27).

We also demonstrated that BECC470s adjuvant significantly enhanced stalk-specific antibody responses against conserved HA domains, promoting broad epitope recognition across diverse influenza subtypes. Antibodies directed against the conserved stalk region of influenza hemagglutinin can protect against a wide range of viral strains, offering broad protection that could overcome the need for regular vaccine reformulation. Moreover, stalk-targeted antibodies can mediate cross-reactive immunity and contribute to the development of a universal influenza vaccine, which is believed to be key for long-lasting and broad protection against seasonal and pandemic influenza virus strains. Notably, BECC470s-adjuvanted sera demonstrated enhanced binding to both heterologous H5N1 and heterosubtypic H1N1 influenza strains, reflecting robust cross-reactivity and magnitude of the humoral immune response.

Building on BECC470s’ proven immunogenicity and protection in H1N1 models (12–14), our H5N1 challenge study demonstrated comparably robust outcomes with adjuvanted formulations significantly outperforming unadjuvanted controls. BECC470s enables antigen sparing alongside balanced IgG1/IgG2a responses, positioning it as a prime adjuvant candidate for next-generation influenza vaccines that enhance breadth and durability against diverse strains, addressing key limitations of current seasonal platforms. Extending this potential, future H5N1 platforms combining BECC470s with NA-enhancing adjuvants could rapidly induce protection at low doses while broadening coverage across emerging subtypes (36, 37). By amplifying HA- and NA-specific antibodies, optimizing B cell recruitment to conserved and strain-specific epitopes, and refining antigen presentation, these strategies address clade 2.3.4.4b vaccine shortcomings to enable more durable, cross-reactive protection (38).

## MATERIALS AND METHODS

### Mouse immunogenicity and challenge study design

Mice were acclimatized for approximately 5 days prior to the start of experimental procedures. They were randomly assigned to cages (*n* = 5 per cage), with each cage representing a single experimental unit within a vaccination group. Vaccinations were administered intramuscularly (i.m.) on day 0 (prime) and day 14 (boost) using 50 μL of the assigned vaccine. Vaccine solutions were prepared by admixing a determined quantity of rHA (0.05–1 µg) with a fibritin trimerization ectodomain (A/Vietnam/1203/2004) and 50 µg of either BECC470s or PHAD (Avanti, AL, USA). The vaccines were incubated with rocking for 1 h to allow interaction between the antigen and the adjuvants. Control groups received 50 μL of sterile PBS.

Blood samples were collected at days 14 and 28 post-prime. On day 28, mice were challenged intranasally (i.n.) with PR8/H5 influenza virus at a dose of 500 PFU/mL. The PR8/H5 challenge virus was a 6:2 reassortment virus of low pathogenicity avian A/Vietnam/1203/2004, in which the polybasic cleavage site was removed (HALo), and N1 from A/Vietnam/1203/2004 in an A/Puerto Rico/8/1934 backbone. Post-challenge, mice were monitored daily for 14 days for clinical signs and weight loss. Mice exhibiting greater than 30% weight loss relative to their pre-challenge baseline were humanely euthanized by gradual CO2 exposure. All surviving mice were euthanized at day 14 post-challenge.

### Mice and immunizations

All mice were female BALB/cJ mice (Jackson Laboratory, Bar Harbor, ME) aged 6 to 8 weeks at the time of vaccination (*n* = 5–15 mice/group).

### Recombinant proteins

Recombinant HA1 and HA stalk proteins from A/Vietnam/1203/2004, as well as full-length recombinant HA (rHA) proteins from A/Vietnam/1203/2004, A/Indonesia/5/2005, A/California/7/2009, and A/BrevigMission/1/1918, were produced with C-terminal hexahistidine tag as previously described (39). Briefly, Expi293F cells were transiently transfected with plasmids encoding the respective HA constructs and cultured in serum-free Expi expression medium following the manufacturer’s protocol. The rHA proteins used for immunization incorporated a modified transmembrane domain replaced by a heterologous carboxy-terminal trimerization motif (fibritin foldon), whereas proteins employed in ELISAs contained a GCN4 isoleucine zipper trimerization domain derived from *Saccharomyces cerevisiae*. Recombinant HAs were purified using NiNTA chromatography. Proper protein conformation was validated by ELISA using a panel of at least six well-characterized monoclonal antibodies known to bind conformation-sensitive epitopes (e.g., CR9114, CT149 [40, 41]). Protein concentrations were quantified using NanoDrop spectrophotometry.

### ELISA

Enzyme-linked immunosorbent assays (ELISAs) were performed to quantify serum antibodies specific to recombinant hemagglutinin (rHA), following a previously described protocol with minor modifications (12, 14). Ninety-six-well plates (Nunc, USA) were coated overnight at 4°C with rHA containing a GCN4 trimerization domain (2 µg/mL in 0.1 M sodium bicarbonate buffer). Plates were then washed three times with PBS-0.05% Tween-20 and blocked for 2 h at room temperature with blocking buffer consisting of 0.5% nonfat dry milk and 3% goat serum in PBS-T. Serial dilutions of serum samples were added to the plates and incubated for 2 h at room temperature. After washing, horseradish peroxidase (HRP)-conjugated secondary antibodies specific for IgG, IgG1, or IgG2a (1:3,000; Thermo Scientific, USA) were added to separate plates for 1 h at room temperature. Following three additional washes, 100 μL of 3,3′,5,5′-Tetramethylbenzidine substrate (BD Biosciences, USA) was applied, and the reaction was terminated after 5.5 min by adding an equal volume of stop solution (KPL, MD, USA). Antibody titers were determined using GraphPad Prism software (GraphPad Software, USA).

### Peptide microarray

Peptide array for HA (peptide array, influenza virus A/Vietnam/1203/2004 [H5N1] Hemagglutinin Protein—Influenza A virus) was obtained from BEI Resources, NIAID, NIH (Cat# NR-18974). The 93 peptide arrays (12 or 17 mers with 11 mer overlaps) were dissolved at a concentration of 1 mg/mL using manufacturer suggested solvents. Ten or 11 sequential peptides were mixed to create 9 peptide pools. Peptides were covalently linked by the free carboxylic acid group to the amino group of the 96 well plate (Thermo Scientific Covalink NH, F8 Cat#478042) by coupling agent 1-ethyl-3-(3-dimethylaminopropyl) carbodiimide (EDAC, Sigma 341006-5GM). Briefly, pools were diluted with MES buffer (pH 6) at a final concentration of 4 µg/mL. The solution was added into 96 well plates followed by 10 μL of EDAC (10 mg/mL in water). Plates were incubated overnight at room temperature and washed with distilled water. Sera from groups of mice (*n* = 5) were pooled, and immune reactivity was assessed by ELISA as described above. To evaluate the reactivity against individual peptides in the pool, each peptide was diluted in MES buffer and coupled onto plates as described earlier.

### Determination of viral titer using focus-forming unit assay

Mouse lung tissues were homogenized in sterile PBS using a bead mill or tissue homogenizer to generate lung homogenates. Homogenates were clarified by low-speed centrifugation (1,000 × *g* for 10 min) and serially diluted in infection medium. Madin-Darby canine kidney (MDCK) cells were seeded at 5 × 10^4^ cells per well in 96-well plates and grown to confluence. Cells were infected with diluted lung homogenates for 1 h at 37°C and then overlaid with infection medium containing 1% methylcellulose to restrict viral spread. After 24-h incubation at 37°C, cells were fixed in 10% neutral buffered formalin (Sigma), quenched with 50 mM ammonium chloride, and permeabilized with 0.1% Triton X-100 in 1% BSA. After a 10-min block with 1% BSA, cells were incubated with primary antibody (1:10,000) for 1 h, followed by secondary antibody (1:3,000) for 45 min. Nuclei were counterstained with Hoechst (1:3,000). Wells were maintained in PBS for imaging on the Celigo cytometer. Viral foci were detected by immunostaining with a primary antibody against influenza A virus nucleoprotein, followed by a fluorophore-conjugated secondary antibody. Foci were visualized and counted using a fluorescence microscope or imaging cytometer. Virus titers were calculated as focus-forming units (FFU) per gram of lung tissue.

### Microneutralization

The microneutralization assay was performed to measure the neutralizing antibody titers against influenza A virus (IAV) using Madin-Darby canine kidney (MDCK) cells. Serum samples were heat-inactivated at 56°C for 30 min prior to use. MDCK cells were cultured in maintenance medium and trypsinized when approximately 70%–90% confluent. Cells were counted and seeded at 5 × 10^4^ cells per well in 96-well flat-bottom plates. Sera samples in duplicate (starting dilution 1:40) were serially diluted twofold across the plate. Serially diluted samples were incubated with 60 µL of virus dilution (MOI of 0.078 based on FFU titers for PR8/H5) for 1 h at room temperature on a shaker. MDCK cells were then washed once with 100 µL of PBS, and 100 µL of the virus–serum mixture was added to MDCK cells. The cells were incubated in the presence of antibody and virus for 24 h at 37°C. After 24 h, cells were fixed with 10% neutral buffered formalin (Sigma). The readout was performed via immunofluorescence assay (described previously). H5N1 virus microneutralization was performed as previously described (42). Twofold serial dilutions of monoclonal antibodies or IVIG were incubated with 10^3.3^ TCID50 of virus for 1 h at room temperature with gentle rocking and then added to confluent MDCK cells in 96-well plates. After 4 days, wells were scored for cytopathic effect, and the neutralizing titer was defined as the reciprocal of the highest antibody dilution that completely prevented infection; the concentration needed to neutralize 100 TCID50 was calculated from this dilution and the starting antibody concentration. Results were analyzed in Microsoft Excel and GraphPad Prism v10.6.0.

### Plaque reduction neutralization test

MDCK-SIAT1 cells, kindly provided by Dr. Jesse Bloom via Dr. Emily Bruce, were maintained in DMEM (Corning #10-013-CV) with 10% FBS (Sigma-Aldrich #12306C-500 mL) at 37°C and 5% CO₂ and were confirmed mycoplasma-free. For PRNT₅₀, 12 µL of serum was treated with 36 µL of RDE II Seiken (Hardy Diagnostics #370013) for 19 h at 37°C followed by 30 min at 36°C, per manufacturer instructions, and stored at 4°C until use. Twelve-well plates (Costar #3512) were seeded the previous afternoon with 1 mL MDCK-SIAT1 cells at 3.5 × 10⁵ cells/mL. RDE-treated sera were serially diluted 1:2 in DMEM starting at 1:40 in round-bottom 96-well plates, mixed 1:1 with A/Netherlands/602/2009 (H1N1) viral stock (graciously provided to us by Dr. Weina Sun) at ~1,750 PFU/mL (yielding ~35 foci/well), and then incubated for 1 h at 37°C, 5% CO_2_. After incubation, 200 µL of each serum–virus mixture was added to PBS-washed (Corning #21-040-CV) confluent monolayers in 12-well plates and adsorbed for 1 h at 37°C, 5% CO₂ with rocking every 15 min. Wells were then overlaid with 1 mL cellulose overlay and incubated for 48 h. At 72 h postinfection, overlays were removed, cells washed with PBS, fixed in 4% formalin (EKI #4499-GAL) for 20 min, and stained with ~1 mL crystal violet solution for 5 min. Plates were rinsed three times, and plaques were counted manually using ImageJ software (v1.54g) (https://imagej.net/ij/index.html).

Each assay included a neutralizing monoclonal antibody (anti-influenza HA A/CA/04/09 [H1N1] H1CA4F8) starting at 1:100 (BEI resources, #NR-42021 Lot:7001140) as a positive assay control and sera from sham-vaccinated, strain- and sex-matched mice as the negative sera control, as well as plate-level controls (virus-only and media-only wells). For PRNT₅₀ calculations, the median foci count of virus-only wells was defined as 100% uninhibited virus. Percent neutralization at each dilution was calculated as (virus-only median − sample foci)/virus-only median, and values were analyzed using the “PRNT50_Calculator” R script to obtain the trapezoidal area under the percent-neutralization vs log₁₀ dilution curve with bootstrapped 95% confidence intervals. For overlays, 2× MEM was prepared from 10× MEM (Gibco #11430-030), 7.5% sodium bicarbonate (Gibco #25080-094), 1 M HEPES (Gibco #15630-080), Pen/Strep (Gibco #15070-063), GlutaMAX (Gibco #35050-061), and 10% BSA (MP Biologicals #02199896), brought to 500 mL with Milli-Q water, and sterile filtered (Corning #430769). A 2% cellulose stock was made by autoclaving 2 g cellulose (Sigma-Aldrich #435224-250G) in 100 mL Milli-Q water. Cellulose overlay consisted of a 1:1 mix of 2× MEM and 2% cellulose with 1 µL of 1,000 µg/mL TPCK-trypsin (Sigma-Aldrich #T8802-100MG) per mL. Crystal violet stock was prepared from Milli-Q water, 200-proof ethanol (Koptec #V1001), and crystal violet (Sigma #C6158-100G) to 10× and diluted 1:10 in water for the working solution. All analysis code is available in the Frieman laboratory GitHub repository (https://github.com/frieman-lab/riley_2026).

### Lung histology

Mouse lungs were fixed by immersion in 4% paraformaldehyde (PFA) in phosphate-buffered saline (PBS) for a minimum of 48 h. The fixed lungs were subsequently processed by the University of Maryland—Baltimore Histology Core Facility, where they were embedded in paraffin, sectioned into 5 µm slices, and stained with hematoxylin and eosin (H&E). Histological scoring was performed in a blinded manner using a scale from 0 (no inflammation) to 5 (severe inflammation), with interstitial and peribronchiolar inflammation evaluated separately. The final inflammation score was calculated by averaging these values.

### Statistical analysis

GraphPad Prism 10.6.0 was used to perform all statistical comparisons. Differences among treatment groups were analyzed as mentioned in the figure legends. Exact *P*-values are given in figure legends, but in general, a *P*-value of less than 0.05 was considered significant for all comparisons. *P < 0.05; **P < 0.001.

## Data Availability

All data are available in the main text or the supplemental material; further inquiries can be directed to the corresponding author.

## SUPPLEMENTAL MATERIAL

Supplemental Figures msphere.00171-26-s0001.pdf Fig. S1 to S4.

ASM does not own the copyrights to Supplemental Material that may be linked to, or accessed through, an article. The authors have granted ASM a non-exclusive, world-wide license to publish the Supplemental Material files. Please contact the corresponding author directly for reuse.
