# Supplementary material for: Optimizing an avian influenza vaccine using a novel Bacterial Enzymatic Combinatorial Chemistry (BECC) TLR4 adjuvant
Source: mSphere. 2026 Jul 10;11(7):e00171-26. doi: 10.1128/msphere.00171-26 (PMC13410986; doi:10.1128/msphere.00171-26)
Supplement: Supplemental Figures — Fig. S1 to S4. [file msphere.00171-26-s0001.pdf]

Supplementary Figures

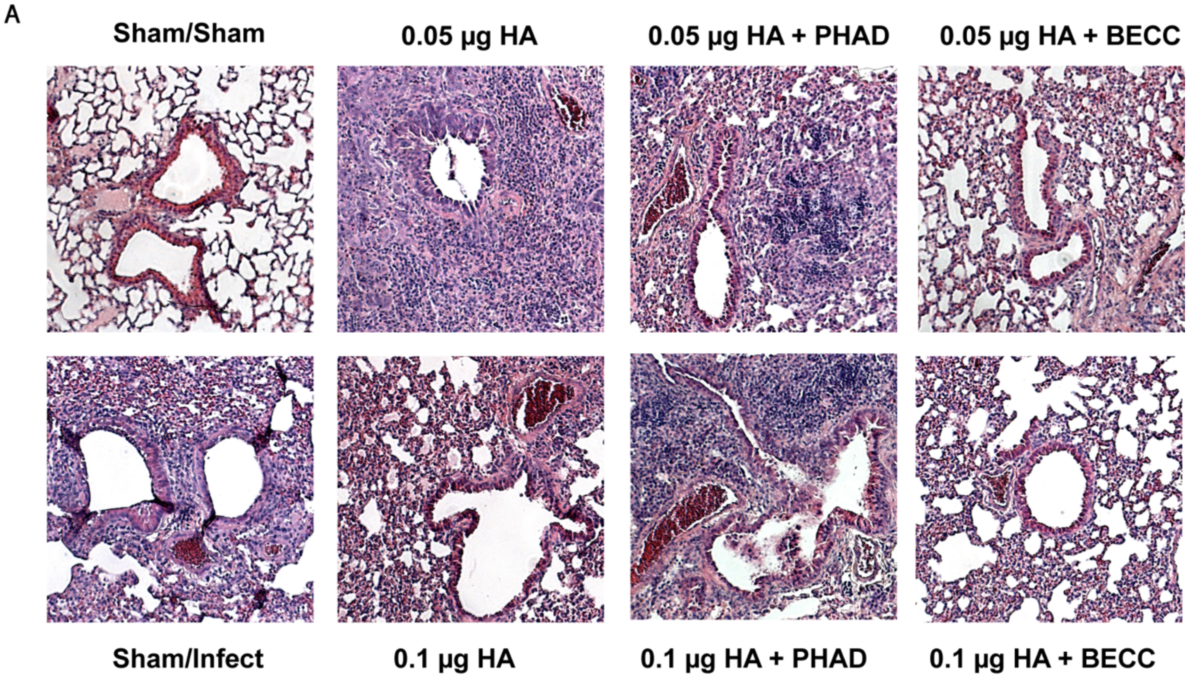

Fig. S1. Histological H&E staining of murine lungs following homologous IAV challenge. Six-week-old BALB/c mice (n = 15) were immunized on days 0 and 14 with 0.05 or 0.1 µg rHA derived from A/Vietnam/1203/2004, formulated without adjuvant, with 50 µg PHAD, or with 50 µg BECC470s, and challenged on day 28 with 500 PFU PR8/H5. Representative lung H&E images are shown at day 14 post-infection for vaccinated groups; for the sham/infected group, images are from day 6 post-infection as no animals survived to the day 14 timepoint.

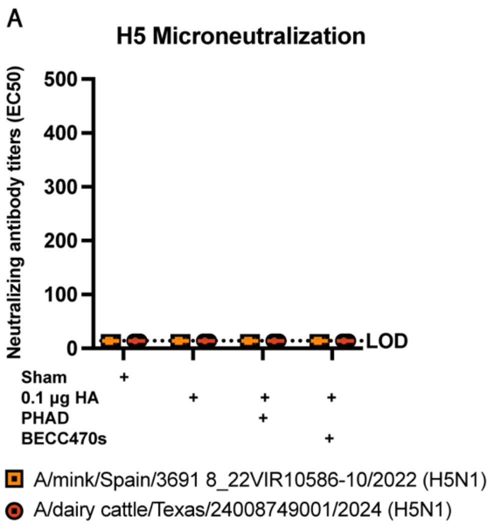

Fig. S2. Neutralization against heterologous H5N1. Neutralizing activity was evaluated by microneutralization assay against A/mink/Spain/3691 8_22VIR10586-10/2022 (H5N1; light brown square) and A/dairy cattle/Texas/24008749001/2024 (H5N1; dark brown circles) viruses. The horizontal dashed line denotes the assay limit of detection (LOD).

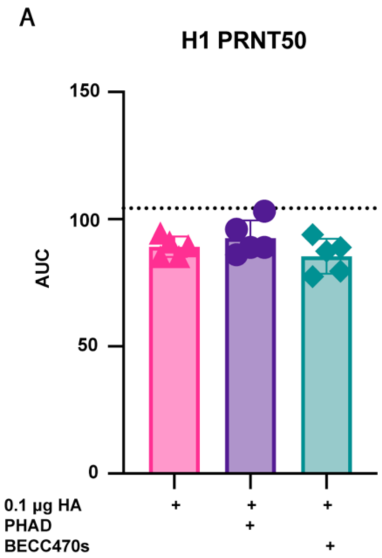

Fig. S3. Neutralization against heterosubtypic H1N1. Neutralizing activity was evaluated by a plaque reduction neutralization test against A/Netherlands/602/2009 (H1N1) virus. The area under the curve (AUC) was calculated for each biological replication (n = 5). The top of the bar represents the condition mean. Error bars are standard deviation. The dashed line represents the average AUC for saline/sham mice (value of 104.3, n=20), which we are considering our negative control samples.

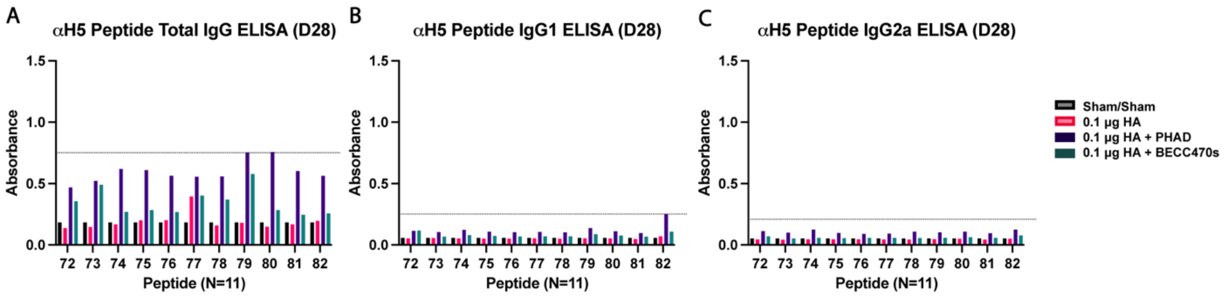

Fig. S4. Linear B cell epitope identification, peptide pool 8 Six-week-old BALB/c mice were immunized via a prime-boost regimen with 0.1 µg rHA derived from A/Vietnam/1203/2004, formulated either without adjuvant, with 50 µg PHAD, or with 50 µg BECC470s. Initially, 93 overlapping peptides (12- or 17-mers with 11 amino acid overlaps) spanning the H5 HA of A/Vietnam/1203/2004 were pooled into nine pools. Pre-infection sera, Day 28 post-immunization, was collected and Total IgG **(A),** IgG1 **(B)** and IgG2a **(C)** titers against peptides 72-82 pools were determined by ELISA. The dashed line indicates an O.D. value fourfold higher than that of the sham control group; samples above this threshold are considered positive.
